# Acute effects of high intensity training on cardiac function: a pilot study comparing subjects with type 2 diabetes to healthy controls

**DOI:** 10.1038/s41598-022-12375-2

**Published:** 2022-05-17

**Authors:** Henning O. Ness, Kristine Ljones, Randi H. Gjelsvik, Arnt Erik Tjønna, Vegard Malmo, Hans Olav Nilsen, Siri Marte Hollekim-Strand, Håvard Dalen, Morten Andre Høydal

**Affiliations:** 1 Department of Circulation and Medical Imaging, Faculty of Medicine and Health, Norwegian University of Science and Technology, Prinsesse Kristinas gt. 3, Akutten og Hjerte-lunge-senteret, 3.etg, 7030 Trondheim, Norway; 2 Clinic of Cardiology, St. Olavs University Hospital, Trondheim, Norway; 3 Department of Medicine, Levanger Hospital, Nord-Trøndelag Hospital Trust, Levanger, Norway; 4 Department of Neuromedicine and Movement Science, Norwegian University of Science and Technology, Trondheim, Norway

**Keywords:** Medical research, Physiology, Cardiovascular diseases, Cardiology, Cardiovascular biology

## Abstract

This study evaluated acute cardiac stress after a high-intensity interval training session in patients with type 2 diabetes (T2D) versus healthy controls. High intensity aerobic exercise was performed by 4 × 4-min intervals (90–95% of maximal heart rate), followed by a ramp protocol to peak oxygen uptake. Echocardiography was performed before and 30 min after exercise. Holter electrocardiography monitored heart rhythms 24 h before, during, and 24 h after the exercise. Left atrial end-systolic volume, peak early diastolic mitral annular velocity, and the ratio of peak early to late diastolic mitral inflow velocity were reduced by approximately 18%, 15%, and 31%, respectively, after exercise across groups. Left ventricular end-diastolic wall thickness was the only echo parameter that significantly differed between groups in response to exercise. The T2D group had a rate of supraventricular extrasystoles per hour that was 265% greater than that of the controls before exercise, which remained higher after exercise. A single exhaustive exercise session impaired left ventricular diastolic function in both groups. The findings also indicated impaired right ventricular function in patients with T2D after exercise.

ClinicalTrials.gov Identifier: NCT02998008.

## Introduction

Type 2 diabetes (T2D) is an increasingly prevalent condition worldwide and is likely to reach pandemic levels within the coming decades^1^. The condition is characterized by high blood glucose and abnormal insulin regulation and is strongly linked to obesity and inactivity. Several complications are associated with the disease, such as kidney disease, retinopathy, cardiovascular disease, and diabetic cardiomyopathy^2^. Low cardiorespiratory fitness (maximal oxygen uptake; VO_2max_), one of the strongest predictors of cardiovascular death, is common in patients with T2D^3^, who are more prone to arrhythmias and their complications such as an increased risk of stroke in those with atrial fibrillation^3^, and cognitive impairment in older adults^4^.

Clinical studies associated diabetes with left ventricular (LV) dysfunction independent of other heart diseases^1^. Myocardial metabolism depends on a sufficient energy substrate provided by fatty acids and glucose during physical exercise. There is a fine-tuned balance in healthy individuals with a dynamic variation in the energy source that depends on the heart’s workload and the exercise intensity^5^. However, this substrate flexibility is significantly impaired in patients with T2D due to insulin deficiency. Therefore, the metabolism depends more on the oxidation of fatty acids, reducing cardiac efficiency^6–8^. Several studies have reported that efficiency of diabetic hearts is further reduced, which cannot be explained by only the reduced substrate flexibility^7,9^. This reduction is ascribed to a certain extent to increased oxygen consumption by non-contractile processes, such as increased mitochondrial uncoupling, production of reactive oxygen species^10,11^, impaired excitation–contraction coupling and Ca^2+^ handling^12,13^. These changes can be prevented by exercise training^14,15^.

Despite numbers of studies showing beneficial effects by exercise, there are also studies showing impairment of the myocardium after vigorous prolonged exercise. A study from the North See Race showed elevated troponin levels after prolonged vigorous exercise among well-trained athletes^16^. There are increasing reports of elevated cardiac troponin (cTn) I and T (TnT) levels, reflecting myocardial cell damage^17^, after exercise interventions. Studies have reported reduced contractility, increased cardiac injury biomarkers and reduced right ventricular (RV) and LV ejection fraction (EF) after long periods of high-intensity training^18–24^. In most cases, the effects are transient; however, persistent changes such as the development of fibrosis have occurred in patients exposed to extreme amounts of training combined with a predisposition for cardiac dysfunction^20^.

## Aim of the study

There is still much to learn regarding the heart’s response to exercise, both in individuals with T2D and those predisposed to ischemic heart disease. The present study aimed to investigate cardiac function and volume responses, biomarkers of cardiac stress, and arrhythmias following a session of high-intensity exhaustive exercise in patients with T2D compared with healthy individuals. We hypothesized that acute exercise training could provoke greater cardiac stress that would manifest with more pronounced cardiac dysfunction in patients with T2D than in healthy individuals. We expected both groups to have elevated TnT levels and indications of impaired cardiac function following exhaustive exercise, with potentially more prominent findings in the RV.

## Results

### Physical characteristics and baseline blood parameters

The mean age was 56 years in both groups. Except from glycated hemoglobin (HbA1c), blood glucose, and insulin C peptide levels, there was no difference between T2D and healthy controls in physical characteristics or blood parameters at baseline (Table 1). In the T2D group, HbA1c levels were significantly higher (33%), glucose levels were 57% higher, and insulin C peptide levels were 50% higher. No other baseline parameters differed between the groups at baseline.

**Table 1:** Physical characteristics and baseline blood samples presented as mean ± standard deviation.

Variable	T2D group (n = 7)	Control group (n = 7)	p
Age (years)	55.9 ± 10.9	56.1 ± 10.9	0.78
Height (cm)	177.7 ± 8.2	183.1 ± 6.0	0.2
Body weight (kg)	87.9 ± 19.3	90.7 ± 9.5	0.64
Skeletal muscle mass (kg)	37.7 ± 7.3	38.8 ± 4.6	0.8
Body fat (%)	25.2 ± 5.3	22.9 ± 8.2	0.62
Body fat (kg)	22.1 ± 7.9	21.9 ± 8.7	0.8
Visceral fat area (cm^2^)	103.3 ± 29.6	101.3 ± 38.3	0.92
In body health score (points)	77.1 ± 5.6	76.4 ± 10.7	0.92
Body mass index (kg/m^2^)	28.0 ± 5.0	27.0 ± 2.8	0.73
Blood pressure systolic (mmHg)	138 ± 12	139 ± 21.2	0.78
Blood pressure diastolic (mmHg)	88.6 ± 11.2	88.9 ± 15.1	0.98
HbA1c (%)	6.9 ± 1.1	5.2 ± 0.2	0.0006
Glucose (mmol/L)	8.6 ± 2.9	5.4 ± 0.3	0.0006
Insulin C peptide (pmol/L)	0.9 ± 0.2	0.6 ± 0.2	0.05
LDL cholesterol (mmol/L)	2.9 ± 0.9	3.7 ± 0.5	0.12
Total cholesterol (mmol/L)	4.7 ± 1.0	5.5 ± 0.7	0.13

*LDL* low-density lipoprotein, *p* p value.

### Response to cardiopulmonary exercise test (CPET)

**Figure 1:**
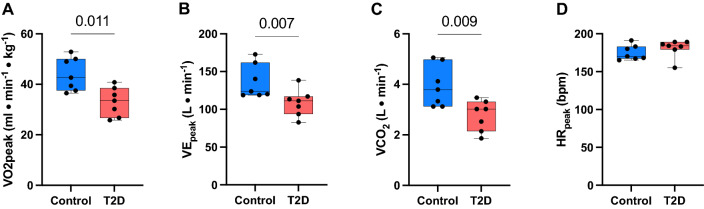
Baseline data from cardiopulmonary exercise tests between the controls and type 2 diabetes (T2D) group. (**A**) Peak oxygen uptake (VO_2peak_) displayed in ml min^–1^ kg^–1^; (**B**) minute ventilation peak (VE_peak_) displayed in L min^−1^; (**C**) ventilatory CO_2_ per minute (VCO_2_) displayed in L min^−1^; (**D**) peak heart rate (HR_peak_) displayed in beats per min (bpm). Data presented with box and whiskers with minimum to maximum values. p values are indicated in figure. Control: n = 7, T2D: n = 7.

VO_2peak_ was significantly lower in the T2D group compared to controls (33.1 ± 5.8 ml kg^−1^ min^−1^ and 44.0 ± 6.6 ml min^–1^ kg^–1^, respectively, p < 0.01) (Fig. 1A). Minute ventilation (VE) and the ventilatory CO_2_ per minute (VCO2) was also significantly reduced in the diabetes group compared to the controls (Fig. 1B,C). The peak heart rate was similar in the two groups with 181 ± 12 beats min^−1^ in the T2D group and 175 ± 10 beats min^−1^ in the control group (Fig. 1D).

### Change in blood samples after exercise

Despite the individual changes observed in several of the measured blood parameters, there were no statistical significant differences in response to exercise between the groups.

### Glucose

**Figure 2:**
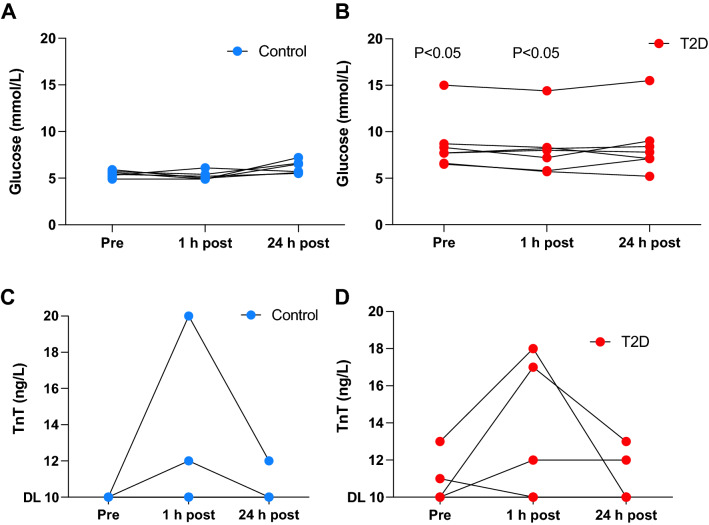
Blood sample data from baseline (Pre) and 1 h and 24 h post-exercise. (**A**) Glucose in the control group; (**B**) glucose in the T2D group; (**C**) Troponin T (TnT) in the control group; (**D**) TnT in the T2D group. Presented with individual data at each time-point. The detection limit for TnT was 10 ng/L. p values between T2D and control are indicated. Control: n = 7, T2D: n = 7.

Glucose levels were expected to decrease in the T2D group following exercise but remained constant (8.6 mmol/L pre-exercise, 8.2 mmol/L post-exercise and 8.6 mmol/L 24 h post-exercise). In the control group, the glucose levels were significantly lower than those in the T2D group but did not change over the different time points (5.4 mmol/L pre-exercise, 5.3 mmol/L post-exercise and 6.2 mmol/L 24 h post-exercise (Fig. 2A,B).

### Troponin T

All of the control group participants had TnT values < 10 ng/L before the exercise training. Two participants of the T2D group had higher values (13 ng/L and 11 ng/L), while the rest had values < 10 ng/L. Three participant in the T2D group had TnT ≥ 10 ng/L after the exercise. Two participants in the T2D group had serum TnT levels that remained elevated after 24 h. Two participants in the control group had elevated TnT levels exceeding 10 ng/L post-exercise; the participant with the highest measured TnT level (20 ng/L) remained above 10 ng/L 24 h post-exercise (Fig. 2C,D).

### Baseline cardiac function in T2D and controls

The T2D group had smaller mean baseline RV end-diastolic diameter (36.4 ± 5.9 mm for T2D vs. 45.9 ± 4.2 mm for control; p < 0.01 (Table 2). There were no other statistically significant differences between the two groups at baseline (before exercise training).

**Table 2:** Echocardiography alterations by exercise training.

Variable	Control group (n = 7)				T2D group (n = 7)				
	Pre	Post	Change	P	Pre	Post	Change	P	
**LV**									
Intraventricular septum thickness, end-diastolic (mm)	8.8 ± 1.0	10.2 ± 0.9	1.5	0.09	10.3 ± 2.1	10.0 ± 1.5	−0.3	0.9	#
LV internal dimension, end-diastolic (mm)	49.1 ± 4.1	48.4 ± 4.1	−0.7	0.44	45.7 ± 4.7	43.7 ± 4.4	−1.9	0.31	
LV posterior wall thickness, end-diastolic (mm)	9.2 ± 1.3	10.1 ± 0.9	0.9	0.02	9.7 ± 1.7	9.5 ± 2.0	−0.2	0.77	
LV fractional shortening (%)	27.1 ± 4.0	29.6 ± 5.5	2.6	0.69	26.1 ± 4.4	27.1 ± 5.1	1.0	0.98	
LV ejection fraction, (%)	59.6 ± 5.3	56.2 ± 3.9	−3.4	0.3	61.7 ± 5.7	58.2 ± 6.7	−3.4	0.16	
LV end-diastolic volume, (ml)	125 ± 14	114 ± 19	−11.4	0.13	106 ± 17	96 ± 16	−10.1	0.16	
Peak systolic mitral annular velocity, mean six walls (cm/s)	7.4 ± 1.0	7.1 ± 1.2	−0.3	0.31	7.2 ± 1.8	7.5 ± 1.5	0.2	0.9	
Peak early diastolic mitral annular velocity (e’), mean six walls (cm/s)	7.7 ± 2.0	6.3 ± 1.9	−1.4	0.02	6.8 ± 1.4	6.0 ± 1.7	−0.8	0.046	
Mitral inflow peak early diastolic (E) velocity (cm/s)	66.2 ± 13.6	52.6 ± 12.7	−13.6	0.03	69.0 ± 11.8	56.8 ± 6.8	−12.2	0.1	
Mitral inflow early diastolic deceleration time (ms)	217 ± 38	338 ± 129	120	0.08	232 ± 55	281 ± 108	50	0.22	
E/A ratio	1.4 ± 0.3	1.0 ± 0.2	−0.4	0.05	1.2 ± 0.5	0.8 ± 0.2	−0.4	0.016	
E/e’ ratio	7.5 ± 2.6	7.0 ± 3.0	−0.5	0.5	8.4 ± 2.3	8.7 ± 3.4	0.3	0.93	
**RV**									
RV basal end-diastolic diameter (mm)	45.9 ± 4.2	43.5 ± 3.4	−2.4	0.16	36.4 ± 5.9	34.9 ± 6.5	−1.5	0.69	$
RV mid-ventricular end-diastolic diameter (mm)	30.1 ± 3.4	28.2 ± 3.0	−1.9	0.39	30.9 ± 1.3	26.5 ± 4.0	−4.4	0.09	
TAPSE (mm)	28.0 ± 4.2	24.7 ± 3.0	−3.3	0.31	23.4 ± 4.3	21.3 ± 1.8	−2.2	0.047	
Tricuspid annular peak early diastolic velocity (cm/s)	9.9 ± 2.3	8.6 ± 1.3	−1.3	0.08	8.3 ± 1.9	6.5 ± 1.9	−1.8	0.016	
**LA**									
LA end-systolic volume (A-L method) ml	74 ± 15	60 ± 19	−13.9	0.016	62 ± 20	51 ± 16	−10.8	0.016	
**RA**									
RA end-systolic volume (A-L method) ml	58 ± 19	62 ± 18	3.4	0.61	42 ± 17	37 ± 15	−4.5	0.16	

Data are mean ± SD. All presented tissue Doppler velocities are recorded by color tissue Doppler, except for E/e’ ratio, which includes the average of septal and lateral e’ measured in pulsed-wave tissue Doppler recordings.

*E/A ratio* peak early mitral inflow to late diastolic velocity, *E/e’ ratio* peak early mitral diastolic inflow velocity (E) to early diastolic mitral annular velocities (e’), *A-L* area-length, *TAPSE* tricuspid annular plane systolic excursion, *LA* left atrium, *RA* right atrium, *LV* left ventricle, *RV* right ventricle.

The indicated p-value is the statistical difference between pre- and post-exercise values within each group. ^#^Significantly different responses from pre- to post-exercise between the T2D and control groups (p < 0.05). ^$^Significant difference between T2D and control groups at baseline recordings before exercise (p < 0.01).

### Cardiac responses to exhaustive exercise in T2D and controls

The only parameter with a statistically significant change from baseline to after exhaustive exercise between groups was end-diastolic intraventricular septum thickness (1.5 mm increase in controls, 0.3 mm reduction in the T2D group; p < 0.05).

Despite the limited intergroup differences in response to exercise, there were statistically significant changes within each groups in several echocardiography parameters (Table 2).

There was a statistically significant reduction in early diastolic mitral annular velocity (e´), reaching 1.4 cm/s in the control group (p < 0.05) and 0.8 cm/s in the T2D group (p < 0.05) when measured by color tissue Doppler, with the pulsed-wave tissue Doppler measurements showing similar findings (Appendix Table A.1). The peak systolic mitral annular velocity was not significant different. The corresponding mitral inflow peak early diastolic velocity (E) was lower after exercise in both groups (− 13.6 cm/s in the control group [p < 0.05] and − 12.2 cm/s in the T2D group [p = 0.1]).

The LV end-diastolic posterior wall thickness increased in the control group after exercise (p < 0.05), while no significant change appeared in the T2D group. A non-significant post-exercise reduction in LV end-diastolic volume compared with baseline was observed in the controls (− 11.4 ml, p = 0.13) and T2D group (− 10.1 ml, p = 0.16). A similar finding was present when analyzing three-dimensional recordings (Appendix Table A.2 and A.3).

There were no differences in the two groups in systolic fractional shortening after exercise. LV EF was > 3.4%-points lower in both groups after exercise, but the differences were not statistically significant. However, in the patients with elevated TnT, LV EF was reduced by 6.7% after exercise (p < 0.05). The early to late peak diastolic mitral inflow velocity (E/A ratio) was significantly reduced by 0.4 in both groups post exercise (p < 0.05).

As a measure of RV function, the tricuspid annular plane systolic excursion (TAPSE) was − 2.2 mm lower in the T2D group (p < 0.05) and − 3.3 in the control group (not statistically significant). Peak early diastolic tricuspid annular velocity was also reduced from baseline to post-exercise in the T2D group (− 1.8 mm, p < 0.05) but was not significantly changed in the control group (− 1.3 mm, p = 0.08).

The left atrial end-systolic volume was significantly reduced from baseline to post-exercise in the T2D group (− 10.8 ml, p < 0.05) and controls (− 13.9 ml, p < 0.05). RV echocardiography detected no changes in either group.

Heart rate during echocardiography did not differ significantly between the groups before or after exercise, but it was significantly higher in the control group during the post-exercise testing (65 ± 13 and 83 ± 14 bpm [p < 0.05] and 69 ± 12 and 79 ± 8 bpm for the controls and T2D group, respectively).

Interestingly, sub-study analyses on echocardiography recordings performed on individuals with post-exercise elevated TnT (independent of the study group) displayed more significant post-exercise deterioration on cardiac function (Appendix Table A.4).

### Holter electrocardiogram

**Figure 3:**
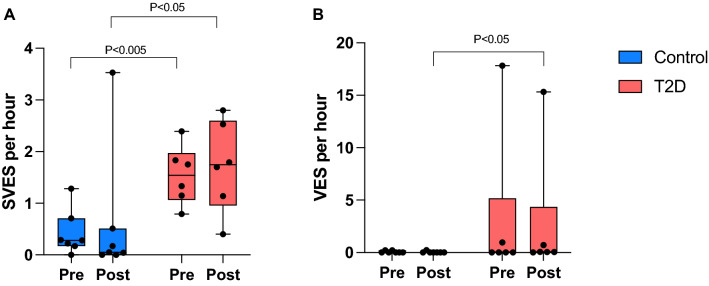
Holter electrocardiography. (**A**) Supraventricular extrasystoles (SVES) per hour (Control: n = 7, T2D: n = 7) and (**B**) ventricular extrasystoles (VES) per hour (Control: n = 7, T2D: n = 6). Data presented with box and whiskers with minimum to maximum values. Pre represents the complete period 24 h before exercise and Post represents the complete period 24 h after termination of exercise. No statistical differences within groups from pre- to post-exercise was observed. p values are indicated.

There were significantly more premature supraventricular extrasystoles (SVES) and ventricular extrasystoles (VES) in the T2D group at baseline (Fig. 3). The T2D group also exhibited more premature ventricular beats after exercise; however, the exercise did not the number of premature beats.

## Discussion

The present study demonstrated statistically significant differences in cardiorespiratory fitness level between the T2D group and controls; however, there were no consistent differences in the cardiac response to exercise training. Interestingly, after exhaustive exercise, both groups had significantly altered e’, E/A ratios, and left atrial end-systolic volumes. These findings indicate that a single session of exhaustive high intensity exercise training affects the hearts of patients with T2D and healthy controls, causing at least intermittent impaired function.

We found that e’ and the E/A ratio were significantly lower in both groups from baseline to post-exercise. Therefore, these changes indicate impaired LV diastolic function after exhaustive exercise in both groups. Diastolic dysfunction refers to disturbed LV relaxation^25^. Similar findings in terms of E/A ratio have occurred after marathon participation with much longer duration^26–28^. Acute adaptations, indicating post-exercise cardiac dysfunction, is further supported by increased LV end-diastolic posterior wall thickness (p = 0.02) and somewhat increased end-diastolic intraventricular septum thickness (p ≤ 0.09) in the control group. This change might reflect post-exercise myocardial edema or increased blood volume in the myocardium. Exercised-induced muscle damage in other muscles has shown an inflammation-like response after exercise, and the finding could indicate a similar mechanism in the myocardium^29^.

Interestingly, these alterations did not appear in the T2D group. The reason for the change in the parameters in the control group and not in the T2D group remains unclear and needs further investigation. However, the lower mitral blood flow velocity (E) and smaller left atrial volumes could indicate that e’ and E/A changes might be partly due to a reduced post-exercise preload.

Peak early diastolic tricuspid annular velocities and TAPSE were reduced after exercise but were only statistically significant in the T2D group, which might indicate RV dysfunction. Based on previous findings, we expected more pronounced changes in RV after exhaustive exercise^18,19,30,31^. However, our results did not indicate more severe changes in the RV than in the LV. This finding might be partly because of the relatively short duration of the exercise training. Although conducted at high intensity, the duration was short compared to previous reports^18,19,31^. Exercise duration has been linked to the degree of RV impairment, where longer durations appear to cause greater impairment^31^. The study by La Gerche et al. hypothesized that long-term processes such as fibrosis might contribute to the development of RV dysfunction^31^.

The acute changes observed after exercise training at high intensity are normally transient. La Gerche found a transient increase in RV volume and a decline in RV function after three to 11 h of exercise, and most patients recover within a week^20,31^. Unfortunately, we did not follow up our population with echocardiograms 24 h after exercise. However, our data showing a decline of TnT levels 24 h after exercise could indicate that the modest systolic and diastolic dysfunction is restored within a relatively short time.

Several studies have reported increased cardiac troponin levels after short and long exercise sessions^32–34^. There is a broad agreement that cardiac impairment following exercise training is greatest in the least trained^22,35^. The T2D group had a statistically significant 25% lower VO_2peak_ than the age-matched controls, which corresponds to previously reported values of cardiorespiratory fitness in this patient group^36^. This reduction in cardiorespiratory fitness has been associated with a two-fold increased risk of cardiovascular mortality among patients with T2D^37^. Myers et al. showed a 12% increase in survival with a 1 metabolic equivalent (~ 3.5 ml kg^−1^ min^−1^) improvement in VO_2peak_^38^. The present study found a substantial ~ 3 metabolic equivalent (11 ml kg^−1^ min^−1^) difference. The difference in cardiorespiratory fitness was further supported by the echocardiographic findings of numerically smaller LVs and RVs in patients with T2D. A greater increase in TnT would be expected in the T2D group because their VO_2peak_ was significantly lower than the controls, demonstrating that the patients with T2D had a significantly lower fitness level. In the North Sea study, a TnT increase was also associated with a higher load of occult obstructive coronary artery disease^39^. Since the T2D group displayed lower aerobic capacity, reduced cardiac function, and a higher load of cardiovascular risk factors than the control group, we wanted to determine whether the T2D group had a more severe post-exercise increase in TnT.

In contrast to our assumption, only a few of the included patients presented with elevated TnT post-exercise. There were no differences between the patients with T2D and the healthy controls. The previously mentioned studies detected increases far above 14 ng/L, which is the cut-off for myocardial infarction^17^. The discrepancy between our results and those of other studies might be due to the differences in exercise duration and total workload over time, with previous studies employing relatively high-intensity exercise over hours. A recent study showed that troponin elevation is probably more closely linked to intensity than duration^40^. A plausible explanation for our findings is that both groups were exposed to the same relatively high-intensity exercise. Notwithstanding, there was an increase in TnT in five participants, three of whom were in the T2D group. Interestingly, those with elevated TnT (independent of group) had more severely impaired cardiac function indications. Several authors suggested that increased troponin levels after exercise could be caused by a physiological response that causes leakage of the unbound free form of troponins from the cytosol during exercise due to membrane damage, not necrosis, including destruction of the contractile apparatus that is common after myocardial infarction^41^.

There were significantly more premature SVES and VES in the T2D group at baseline, agreeing with previous related studies. That might result from changes caused by T2D and the effects of common comorbidities such as hypertension on the heart and autonomic nervous system^42,43^. There was no further effect of exercise on number of premature beats.

T2D is characterized by hyperglycemia, commonly diagnosed by elevated HbA1c levels. Hyperglycemia is a significant risk factor for cardiovascular disease. Engaging in exercise training improves glucose uptake and insulin sensitivity for several hours, depending on the exercise intensity and duration^44^. The exercise intensity determines the use of substrates available during exercise. Higher intensities depend on utilizing glucose, while up to approximately 60% intensities result in greater fat oxidation^45^, which could be important for the glucose regulation commonly observed after exercise. Postprandial glycemic function has been reported to improve acutely after exercise, and high-intensity exercise has been reported to have greater and more lasting effects^46–48^. However, we did not use continuous glucose monitoring in our study and cannot confirm nor exclude similar results. There was no change in glucose after exercise in any of the groups. HbA1c was measured only during screening to confirm the presence of T2D.

### Study strengths and limitations

The main limitation of this pilot study is the small sample size, and the results obtained should be considered carefully and serve as a fundament for further studies. The patients with T2D also presented with relatively mild symptoms of diabetes. This population typically has body mass indices (BMI) > 30, while our participants had a mean BMI of 28. CPET displayed the largest differences between the groups. Before the study, there were doubts about whether the T2D group would be able to perform maximal effort during CPET. However, there was no significant difference in the respiratory exchange ratio between the controls and the T2D group. Also, the echocardiographic examinations were performed at rest, and stress echocardiography might have been a better-suited measure to determine cardiac performance differences. The blinding for group assignment and time in the echocardiographic analyses constitutes one of the study’s strengths. The highly relevant methods for exploring acute responses to exhaustive exercise in patients with T2D and healthy individuals constitute another of the study’s strengths.

## Conclusion

The present study found no major differences in the cardiac response to acute exhaustive exercise between the patients with T2D and the healthy controls. After exercise training, both groups had reduced cardiac function, displayed by reduced e’ and the E/A ratio, indicating LV diastolic function. Following exercise, there were no consistent differences between the T2D and control groups in cardiac troponin release. Despite the significantly greater number of premature SVES and VES in the T2D group than in the control group, exercise did not alter the electrophysiological parameters from baseline. The comprehensive and blinded echocardiographic and electrophysiological analyses provided valuable information regarding the acute cardiac responses to high-intensity training in patients with T2D versus healthy controls and provided hypotheses for future research.

## Methods

### Subjects

The study included age-matched subjects with T2D (n = 7) and healthy controls (n = 7). In total, 15 men volunteered for the study; one person was excluded due to a lack of parameters confirming T2D. Inclusion criteria were an age > 30 years and confirmed T2D accompanied by elevated HbA1C and glucose levels. The patients with T2D were on optimal medication advised from their medical doctors (Individual medications and history of T2D included in Appendix Table A.5) None of the T2D presented with diabetic complications (retinopathy, nephropathy, neuropathy, etc.). All participants provided written informed consent before data collection. The Regional Committee for Medical Research Ethics Central Norway approved this study (REC ID 2016/1596), which complied with the statement of ethical principles for medical research outlined in the Declaration of Helsinki. All methods were performed under the relevant guidelines. The study is registered in ClinicalTrials.gov Identifier: NCT02998008 (First posted: 20/12/2016).

## Test procedure and protocols

### Participant registration and timeline

#### Day 1

All participants completed a questionnaire from a physician evaluating their health status and medical consent for training and CPET. Blood pressure was measured following 10 min of rest while seated in a chair. Three tests were performed and the mean result of the latter two was used. The participants had their height measured in cm and then underwent an Inbody scan (Inbody, AU). They subsequently underwent Holter electrocardiogram (ECG) monitoring of the cardiac electrical activity 24 h before, during, and 24 h after the exercise. Lastly, a blood sample was collected from the participants at the end of day 1.

#### Day 2

Echocardiography was first performed immediately before the single session of exhaustive exercise consisting of a 4 × 4-min interval exercise and one final bout performed as a ramp protocol to assess VO_2peak_. After the exercise all participants rested for 30 min before undergoing the second echocardiography. At the end of day 2, a second blood sample was collected 30 min after the second echocardiography.

#### Day 3

Participants concluded their Holter ECG monitoring, and a final blood sample was collected 24 h after the exercise test.

### Interval training and CPET

The exercise was performed as a 4 × 4-min high-intensity interval training. The intervals were conducted at approximately 90% of VO_2peak_ and were followed by 2-min breaks with active recovery at 60%. We then added a fifth interval performed as a ramp protocol with speed and/or inclination increments to measure VO_2peak_. The CPET to measure VO_2peak_ was performed at the NeXt Move core facility at the Norwegian University of Science and Technology. All participants were regularly offered water during and after exercise to prevent hypovolemia. Given that patients with T2D sometimes have physical limitations, experienced personnel determined the best individual CPET regimen during a 6-min warm-up on the treadmill (Woodway PPS55, USA Inc., Waukesha, WI, USA), by detecting functional walking or running speed and inclination, as well as subjective moderate aerobic intensity based on rated perceived exertion (RPE Borg scale 6–20). Participants were then fitted with a heart rate monitor (H7, Polar Electro, Kempele, Finland) and facemask (7450 Series V2 CPET mask, Hans Rudolph Inc., Shawnee, KS, USA). Work economy measurements were performed during an initial 4-min period at a fixed submaximal workload serving as an extended warm-up.

VO_2max_ was defined using the following criteria: (1) VO_2_ levelling off (< 2 ml min^–1^ kg^–1^) despite the increased workload and (2) respiratory exchange ratio ≥ 1.05. If these criteria were not met, the term VO_2peak_ was used. A participants’s VO_2peak_ was defined as the mean of the three successive highest VO_2_ measurements recorded during the CPET. For simplicity, the term VO_2peak_ is used for all patients.

An individualized ramp protocol was used until either exhaustion or fulfilment of the criteria for VO_2max_ or VO_2peak._ The workload was gradually increased, and gas measurements were recorded every tenth second using a mixing chamber ergospirometry system (Metalyzer II, Cortex Biophysik Gmbh, Leipzig, Germany).

### Echocardiography

Echocardiographic recordings and analyses of the different chambers followed the recommendation by the American and European societies of Echocardiography^49^. An experienced cardiologist performed transthoracic echocardiography, and all participants were examined in the left-lateral decubitus position. A Vivid E95 scanner was employed with a phased-array transducer (M5S) (GE Ultrasound, Horten, Norway). Echocardiographic data were stored digitally and analyzed after end of study inclusion by the same cardiologist. All echocardiograms were acquired with the operator blinded to group assignment to avoid any bias in the analyses. All analyses were performed by the same operator blinded to group assignment and whether the echocardiogram was recorded before or after the exhaustive exercise session. All measurements reflect the mean of three cardiac cycles, as recommended for patients in sinus rhythm. The measurements are reported as absolute values and not indexed to body surface area.

Grey-scale two-dimensional views were recorded from the parasternal border in short- and long-axis, and the apical position in four-chamber, two-chamber, and long-axis views. Separate recordings were made to optimize the volumetric measurements of the specific chambers and avoid foreshortening and misalignment. Linear measurements of the LV myocardium and dimensions were done in parasternal long-axis recordings at end-diastole and end-systole immediately below the level of the mitral valve leaflet tips. The fractional shortening was calculated by the change in LV size divided by the end-diastolic size. Left atrial and LV volumes were measured by the summation of discs method in four- and two-chamber views by tracing of the endocardial border. LV EF was calculated as the percentage of blood volume ejected during systole using biplane method of disc summation (Simpson’s method). The area-length method estimated right atrial and RV volumes from RV-focused four-chamber views. The RV dimension was measured in RV-focused four-chamber views at the basal and mid-ventricular levels. TAPSE was measured by reconstructed motion mode aligned to the movement of the basal RV free wall. Recordings were performed in color-coded Doppler mode through all valvular orifices and vessels to identify conditions such as regurgitation and stenosis.

Spectral Doppler with a sample volume recorded blood flow; (a) at tip of the mitral leaflet and in the presence of mitral regurgitation aligned to the regurgitant jet, (b) in the distal LV outflow tract, (c) through the aortic valve, (d) at tip of the tricuspid valve and in presence of tricuspid regurgitation aligned to the regurgitant jet, and (e) in the RV outflow tract. Care was taken to align the ultrasound beam to the blood flow direction for all measurements. The mitral inflow peak early (E) and late (A) diastolic velocities and the early diastolic deceleration time was measured, and the E/A ratio was calculated.

Color tissue Doppler cine-loops were recorded in the apical four-chamber, two-chamber and long-axis, and RV-focused view. Target frame rate for the color tissue Doppler recordings was 100 fps. Care was taken to align the ultrasound beam to the myocardial wall. Peak systolic (S’) and early diastolic (e’) mitral annular velocities were measured at the base of the six myocardial walls by color tissue Doppler, and the average values are used as measurements of the LV myocardial velocities. Pulsed-wave tissue Doppler velocity curves were recorded from the basal part of the left and right ventricle, at the septal and lateral points (near the insertion of the mitral valve) and from the RV free wall (near the insertion of the tricuspid valve). e’ was measured at the base of the septal and anterolateral wall by pulsed-wave tissue Doppler and the average was used for calculation of the E/e’ ratio. The peak systolic and early diastolic tricuspid annular velocities were measured by color tissue Doppler and pulse-wave Doppler, in the basal part of the right ventricular free wall.

### Biochemical analysis

Blood samples were analyzed following standard operating procedures at St. Olavs Hospital. Glucose, HbA1c, total cholesterol, low density lipoprotein LDL cholesterol, TnT and insulin C peptide were all obtained before the training. Glucose and TnT were also obtained 1 h and 24 h post-exercise.

### Body composition and weight

Body composition was measured using the validated bioelectrical impedance unit, Inbody 720 (Biospace, Seoul, Korea)^50^. In this machine, four pairs of electrodes are implanted into the handles and floor scale of the analyzer. Before testing, participants fast for a minimum of two hours and are encouraged to go to the toilet right before using the scale. The participants stood for 5 min before entering the scale and were barefoot. Due to the electrical impulse, people with pacemaker were not tested. Height, age, and sex were plotted om the scale display. After two minutes, weight (kg), BMI, muscle mass (kg), bodyfat percentage, and visceral fat (cm^2^) were measured by the scale. The device was auto calibrated once a week with the machine turned off.

### Heart rhythm

A 48-h ambulatory, continuous ECG recording (DigiTrak XT, Phillips Healthcare, Andover, MA) was used 24 h before, during, and 24 h after the exercise session. Supraventricular and ventricular premature beats and arrhythmias were counted by the vendor-specific software but manually controlled by a trained physician.

### Statistical analysis

A two-tailed Mann–Whitney test (unpaired samples) was applied to compare the changes from baseline to post-exercise between the groups (T2D and control). To compare the changes from baseline to post-exercise within each group, we used a two-tailed Wilcoxon matched-pairs signed-rank test, with exact p values. Heart rhythm measured using Holter electrocardiography monitoring was analyzed by Mann–Whitney two-tailed test (exact p values) at basline and post-exercise. Differences in TnT and glucose levels in the blood samples between T2D and control at baseline and at 1 h and 24 h post-exercise was performed using a mixed-effect model for repeated measures corrected with Šídák's multiple comparisons test. With TnT as a cardiac stress marker, we performed separate sub-analyses to determine the cardiac function response to exercise in individuals increased TnT levels. Data from the individuals with increased TnT levels (i.e., TnT > 10 ng/L) were therefore analyzed as a separate population using two-tailed Wilcoxon matched-pairs signed rank test, with exact p values. All analyses was performed using GraphPad Prism (version 9.3.1). p values < 0.05 were considered significant.

### Ethics approval

The Regional Committee for Medical Research Ethics Central Norway approved this study (REC ID 2016/1596), which complied with the ethical ethical principles for medical research outlined in the Declaration of Helsinki. All methods were performed under the relevant guidelines and in agreement with the approval from the ethical committee. The study is registered in ClinicalTrials.gov Identifier: NCT02998008 (First posted: 20/12/2016).

## Supplementary Information

Supplementary Tables.

## Data Availability

The datasets analyzed during the current study are available from the corresponding author on reasonable request.
