# Supplementary material for: Acute effects of high intensity training on cardiac function: a pilot study comparing subjects with type 2 diabetes to healthy controls
Source: Sci Rep. 2022 May 17;12:8239. doi: 10.1038/s41598-022-12375-2 (PMC9114004; doi:10.1038/s41598-022-12375-2)
Supplement: Supplementary file 1 — Supplementary Tables. [file 41598_2022_12375_MOESM1_ESM.docx]

**Appendix table A1.** Functional measurements of left and right ventricular function.

|  | **Control** | | | | **Diabetes** | | | |
| --- | --- | --- | --- | --- | --- | --- | --- | --- |
| **Variable** | **Pre** | **Post** | **Difference** | **P** | **Pre** | **Post** | **Difference** | **P** |
| RV fractional area change | 0.4 ± 0.1 | 0.4 ± 0.1 | 0.0 | 0.62 | 0.4 ± 0.11 | 0.4 ± 0.1 | 0.0 | 0.75 |
| Tricuspid annular S’ (cTD), cm/s | 12.4 ± 2.5 | 11.8 ± 2.5 | −0.6 | 0.14 | 12.5 ± 2.0 | 11.6 ± 1.7 | −0.9 | 0.32 |
| Tricuspid annular e’ (cTD), cm/s | 9.9 ± 2.3 | 8.6 ± 1.3 | −1.3 | 0.08 | 8.3 ± 1.9 | 6.5 ± 1.9 | −1.8 | 0.016 |
| Tricuspid annular a’ (cTD), cm/s | 10.7 ± 3.1 | 10.2 ± 3.5 | −0.5 | 0.5 | 12.7 ± 2.3 | 12.9 ± 2.2 | 0.3 | 0.94 |
| Tricuspid annular S’ (pwTD), cm/s | 14.5 ± 3.3 | 13.8 ± 2.9 | −0.7 | 0.37 | 14.9 ± 1.8 | 15.4 ± 3.2 | 0.6 | 0.81 |
| Tricuspid annular e’ (pwTD), cm/s | 13.2 ± 3.1 | 10.9 ± 2.6 | −2.3 | 0.047 | 10.1 ± 1.6 | 8.1 ± 1.4 | −2.0 | 0.016 |
| Tricuspid annular a’ (pwTD), cm/s | 14.1 ± 3.7 | 12.1 ± 3.4 | −1.9 | 0.16 | 15.2 ± 2.5 | 15.4 ± 2.9 | 0.3 | 0.94 |
| Mitral annular a’, mean 6 walls (cTD), cm/s | 7.8 ± 1.2 | 8.3 ± 0.7 | 0.5 | 0.38 | 8.8 ± 0.9 | 9.3 ± 1.3 | 0.5 | 0.3 |
| Mitral annular S’, mean septal and lateral walls (pwTD), cm/s | 8.7 ± 1.5 | 8.8 ± 1.9 | 0.1 | 0.92 | 8.8 ± 2.6 | 8.9 ± 2.4 | 0.2 | 0.56 |
| Mitral annular e’, mean septal and lateral walls (pwTD), cm/s | 9.4 ± 2.1 | 8.1 ± 2.0 | −1.3 | 0.047 | 8.8 ± 3.2 | 7.2 ± 2.2 | −1.6 | 0.1 |

Abbreviations: Pre, pre-exercise measurement; Post, post-exercise measurement; RV, Right ventricle; a’, peak late diastolic velocity; cTD, continuous wave Doppler; e’, peak early diastolic velocity; pwTD, pulsed-wave tissue Doppler; S’, peak systolic velocity; P, p-value.

**Appendix table A.2.** Cardiac chamber dimensions.

|  | **Control** | | | | **Diabetes** | | | |
| --- | --- | --- | --- | --- | --- | --- | --- | --- |
| **Variable** | **Pre** | **Post** | **Difference** | **P** | **Pre** | **Post** | **Difference** | **P** |
| RV basal end-diastolic diameter, mm | 45.9 ± 4.2 | 43.5 ± 3.4 | −2.4 | 0.16 | 36.4 ± 5.9 | 34.9 ± 6.5 | −1.5 | 0.69 |
| RV mid end-diastolic diameter, mm | 30.1 ± 3.4 | 28.2 ± 3.0 | −1.9 | 0.39 | 30.9 ± 1.3 | 26.5 ± 4.0 | −4.4 | 0.09 |
| RV longitudinal internal end-diastolic diameter, mm | 74.2 ± 4.5 | 73.6 ± 3.9 | −0.6 | 0.45 | 73.9 ± 10.6 | 71.8 ± 8.1 | −2.1 | 0.58 |
| RVOT distal end-diastolic diameter, mm | 22.5 ± 2.3 | 22.3 ± 1.9 | −0.2 | 0.84 | 21.8 ± 2.6 | 23.1 ± 2.6 | 1.3 | 0.47 |
| RVOT proximal end-diastolic diameter, mm | 33.0 ± 1.2 | 34.1 ± 3.8 | 1.1 | 0.56 | 32.9 ± 5.6 | 34.7 ± 4.3 | 1.8 | 0.3 |

Abbreviations: Pre, pre-exercise measurement; Post, post-exercise measurement; P, p-value; RV, right ventricular; RVOT, right ventricular outflow tract.

**Appendix table A.3.** Cardiac chamber volume measurements

|  | **Control** | | | | **Diabetes** | | | |
| --- | --- | --- | --- | --- | --- | --- | --- | --- |
| **Variable** | **Pre** | **Post** | **Difference** | **P** | **Pre** | **Post** | **Difference** | **P** |
| LA end-systolic volume index, biplane (MOD), ml/m^2^ | 31.3 ± 6.6 | 25.5 ± 6.6 | −5.8 | 0.1 | 28.9 ± 8.8 | 24.1 ± 8.2 | −4.7 | 0.08 |
| LA end-systolic volume index, biplane, (A-L), ml/m^2^ | 34.8 ± 7.7 | 28.6 ± 6.9 | −6.2 | 0.08 | 30.9 ± 9.2 | 26.0 ± 9.1 | −4.8 | 0.08 |
| LA end-systolic volume, biplane (A-L), ml | 74 ± 15 | 61 ± 16 | −12.8 | 0.1 | 64 ± 21 | 54 ± 20 | −10.4 | 0.08 |
| LA end-systolic volume, 4-chamber view only (A-L), ml | 70 ± 16 | 59 ± 17 | −11.0 | 0.1 | 66 ± 26 | 56 ± 27 | −9.9 | 0.3 |
| LA end-systolic volume, 2-chamber view only (A-L), ml | 74 ± 15 | 60 ± 19 | −13.9 | 0.016 | 62 ± 20 | 51 ± 16 | −10.8 | 0.016 |
| LA end-systolic volume, 4-chamber view only (MOD), ml | 63 ± 15 | 52 ± 17 | −10.9 | 0.08 | 62 ± 24 | 51 ± 24 | −10.5 | 0.24 |
| LA end-systolic volume, 2-chamber view only (MOD), ml | 67 ± 12 | 55 ± 17 | −12.4 | 0.2 | 59 ± 19 | 49 ± 16 | −10.2 | 0.016 |
| LA end-systolic length, 4-chamber view, cm | 5.7 ± 0.8 | 5.1 ± 0.6 | −0.5 | 0.06 | 5.5 ± 1.0 | 5.0 ± 0.6 | −0.5 | 0.06 |
| LA end-systolic length, 2-chamber view, cm | 5.5 ± 0.5 | 5.3 ± 0.5 | −0.3 | 0.3 | 5.5 ± 0.9 | 5.1 ± 0.7 | −0.3 | 0.047 |
| RA end-systolic volume (A-L), ml | 58 ± 19 | 62 ± 18 | 3.4 | 0.69 | 42 ± 17 | 37 ± 15 | −4.5 | 0.16 |
| RA end-systolic volume, 4-chamber view (MOD), ml | 55 ± 18 | 58 ± 17 | 4.0 | 0.6 | 39 ± 15 | 35 ± 13 | −4.3 | 0.08 |
| RA end-systolic length, 4-chamber view, cm | 5.1 ± 0.4 | 5.2 ± 0.3 | 0.1 | 0.63 | 5.2 ± 0.7 | 4.9 ± 0.6 | −0.3 | 0.03 |

Abbreviations: A-L, area-length method; LA, left atrium; LV, left ventricle; MOD, summation of discs method; RA, right atrium; P, p-value.

**Appendix Table A.4. Selection of echocardiographic values in individuals with post exercise Troponin T >10 ng/L**

| Variable | Pre | Post | Difference | P |
| --- | --- | --- | --- | --- |
| LV | | | | |
| Intraventricular septal thickness, end-diastolic (mm) | 9.6 ± 2.3 | 10.3 ± 1.7 | 0.7 | 0.4 |
| LV internal dimension, end-diastolic (mm) | 45.7 ± 5.1 | 43.1 ± 5.1 | −2.6 | 0.25 |
| LV posterior wall thickness, end-diastolic (mm) | 10.1 ± 1.6 | 10.5 ± 1.8 | 0.8 | 0.3 |
| Left ventricular ejection fraction (%) | 62.2 ± 5.3 | 55.5 ± 3.0 | −6.7 | 0.03 |
| Left ventricular end-diastolic volume (ml) | 115 ± 22 | 95 ± 16 | −19.8 | 0.03 |
| Peak systolic mitral annular velocity, mean six walls (cm/s) | 6.9 ± 1.3 | 6.8 ± 0.8 | −0.02 | 0.4 |
| Peak early mitral annular diastolic velocity (e’), mean six walls (cm/s) | 6.6 ± 1.8 | 5.7 ± 1.8 | −1.0 | 0.03 |
| Mitral inflow peak early diastolic velocity (E) (cm/s) | 72.6 ± 10.4 | 54.3 ± 11.0 | −18.3 | 0.03 |
| Mitral inflow early diastolic deceleration time (ms) | 224.1 ± 32.6 | 292.8 ± 89.8 | 68.7 | 0.2 |
| E/A ratio | 1.4 ± 0.4 | 0.9 ± 0.2 | −0.5 | 0.03 |
| E/e’ ratio | 9.0 ± 2.6 | 8.8 ± 4.5 | −0.2 | 0.7 |
| RV | | | | |
| RV basal end-diastolic diameter (mm) | 39.2 ± 9.8 | 37.9 ± 9.2 | −1.3 | 0.3 |
| RV mid-ventricular end diastolic diameter (mm) | 30.3 ± 2.9 | 26.9 ± 4.4 | −3.5 | 0.2 |
| TAPSE (mm) | 25.7 ± 5.0 | 21.2 ± 1.6 | −4.5 | 0.03 |
| Tricuspid annular peak early diastolic velocity, color tissue Doppler (cm/s) | 9.3 ± 1.7 | 7.5 ± 1.9 | −1.8 | 0.03 |
| LA | | | | |
| LA end-systolic volume, biplane (ml) | 63 ± 25 | 48 ± 16 | −15.5 | 0.03 |
| RA | | | | |
| RA end-systolic volume (ml) | 48.0 ± 25.1 | 42.6 ± 21.3 | −5.3 | 0.09 |

Data are mean±SD. E/A ratio: Ratio- Mitral inflow peak early to late diastolic velocity; E/e’ ratio: Ratio-Mitral inflow peak early diastolic velocity (E) to early diastolic mitral annular velocities (e’); All presented tissue Doppler velocities are recorded by color tissue Doppler, except for E/e’ ratio which includes the average of septal and lateral e’ measured in pulsed-wave tissue Doppler recordings. A-L method: area-length method. TAPSE: tricuspid annular plane systolic excursion; P, p-value

**Appendix Table A.5 Medication and history of diagnosed T2D.**

| **Group** | **Time with diagnosed T2D (years)** | **Medications** |
| --- | --- | --- |
| T2D | 6 |  |
| T2D | 7 | Eucreas, Metformin |
| T2D | 3 | Atacand plus, Lipitor |
| T2D | 2 | Janumet, Metformin |
| T2D | 10 | Eucreas, Somac, Statin, Albyl-E |
| T2D | 3 | Metformin, Atorvastatin, Valsartan |
| T2D | 10 | Eucreas, Mindiab |
| Control |  |  |
| Control |  |  |
| Control |  |  |
| Control |  |  |
| Control |  |  |
| Control |  | Rhinocort (Allergy) |
| Control |  |  |
